# Opportunities and obstacles to the elimination of malaria from Peninsular Malaysia: knowledge, attitudes and practices on malaria among aboriginal and rural communities

**DOI:** 10.1186/1475-2875-9-137

**Published:** 2010-05-24

**Authors:** Abdulelah H Al-Adhroey, Zurainee M Nor, Hesham M Al-Mekhlafi, Rohela Mahmud

**Affiliations:** 1 Department of Parasitology, Faculty of Medicine, University of Malaya, 50603, Kuala Lumpur, Malaysia

## Abstract

**Background:**

Despite continuous efforts by the government and private sectors, malaria is still a public health problem in rural Peninsular Malaysia. This study investigated household knowledge, attitude and practices (KAP) regarding malaria in two malaria endemic communities, forest-aboriginal and rural communities, in the Lipis district of Pahang state, Malaysia.

**Methods:**

A descriptive cross-sectional study with a semi-structured questionnaire was carried out among 100 and 123 households from forest-aboriginal and rural areas, respectively.

**Results:**

Knowledge about malaria and its transmission is significantly higher among the rural participants than the aborigines (86.2% vs 76%, p < 0.01). However, use of medicinal plants and beliefs in witchcraft and sorcery in treating febrile diseases were significantly higher among the aboriginal population (p < 0.01). There were no significant differences between the two communities in terms of the knowledge about malaria symptoms, attitudes towards its severity and practices in preventive measures against malaria by using mosquito bed nets. However, the knowledge and practice of different preventive measures to combat malaria, such as insecticide and the elimination of breeding areas, was significantly higher among the rural population than the aborigines (p < 0.001).

**Conclusions:**

Both communities were aware of malaria as a disease, but knowledge, attitudes and practices were inadequate. Providing efficient health education to people residing in malaria endemic areas would improve their understanding about malaria prevention in order to bring about the elimination of malaria from the country.

## Background

Malaria, a tropical disease caused by protozoan parasites of the genus *Plasmodium*, is one of the most important infectious diseases in the world. Its global burden and economic cost are still enormous, and it caused about 250 million cases resulting in nearly one million deaths in 2006 [1].

The malaria control programme in Malaysia, established in 1901, has achieved significant success in controlling malaria and a tremendous reduction in the number of malaria cases has been achieved, from about 300,000 cases in 1961 to about 6,000 cases since 2003, and the affected areas have become more and more restricted [2]. However, a sudden increase in the incidence of malaria was reported in 2008 and this may call for an urgent update in the means of eliminating the disease [1]. Even though indoor residual spraying of insecticides is a good method of malaria control in Malaysia [3], the elimination of malaria from a community requires the combination of several measures for the implementation of that strategy. The participation of the community represents one of the cardinal tools of malaria control programmes conducted by the WHO as an improvement in the understanding of the transmission of malaria can greatly increase the realization and sustainability of malaria elimination programmes [4,5].

In parallel with the implementation of Malaysia's malaria elimination programme, this study was conducted to investigate the KAP on malaria transmission, treatment and vector control among the forest-aboriginal and the rural communities in Lipis district, Pahang state, which still represents the area with the highest prevalence of malaria cases in Peninsular Malaysia.

## Methods

### Study area

Lipis district in Pahang state is located 200 km north-east Kuala Lumpur, Peninsular Malaysia with an area of 5,198 km^2^. The climate is equatorial with an average temperature of 23-32°C and an annual rainfall of between 1525-3050 mm. Two rural areas inhabited by the rural population (Padang Tengku and Benta) and one forest area inhabited by the aboriginal peoples (Pos Betau) were involved in this study. The two areas are situated at a distance of about 25-50 kilometres from one another. About 59 small villages (8-40 houses) are the total number of villages in these areas (23 in Padang Tengku, 18 in Benta and 18 in Pos Betau) with approximately 1,000-1,200 households. In terms of the structure of the houses, 90% of the aboriginal houses are made of bamboo and have no toilets while all the houses of the two rural areas are made of cement blocks and have toilets and water supply. The economy is mostly agricultural with palm and rubber plantations occupying the majority of the total land area. Malaria transmission in the area is endemic and varies from one area to another, the highest rate of infection being in the aboriginal area (Pos Betau) followed by the Padang Tengku. The foundations of malaria control are impregnated bed nets, the indoor spraying of insecticide, and early diagnosis and treatment performed by the Vector-Borne Diseases Control Unit in Kuala Lipis, the capital city of Lipis district.

### Study design and data collection

This descriptive cross-sectional survey was conducted between October 2008 and May 2009 among 223 adult households (84 Men and 139 women) who had or had not suffered a malaria crisis. The selection of the participants was universal, according to the availability and willingness of the people. Small community meetings were held before the commencement of the study with the heads of the villages and households in order to give a clear explanation about the objectives of the study and their involvement. Verbal consent was obtained from the participants. The protocol of this study has been approved by the Medical Research Committee of the University of Malaya Medical Centre, and the Department of aboriginal Affairs, Ministry of Rural Development.

Socio-demographic data of the households were collected using a pre-tested semi-structured questionnaire constructed in English and translated into Malay, the local language. The questionnaire, which also involved open-ended questions to investigate the KAP on malaria, was administered to the head of each household by trained assistants from the nearest health centre.

Data were analysed using SPSS version 13.0. The Chi-square test was used to examine the differences between aboriginal and rural participants for each of the outcome variables and p ≤ 0.05 was considered as the level of significance.

## Results

### General characteristics of participants

Out of the 260 households that participated in this survey, questionnaires were only completed for 223 households (100 forest-aboriginal and 123 rural). Table 1 shows the general socio-demographic characteristics of the study population. Overall, 10% of the aboriginal households had suffered from malaria during the last year compared to 1.6% of rural households. Almost half (54.8%) of the aboriginal women had no formal education compared with 10.5% of men (*X*^2 ^= 21.549, p < 0.001). In contrast, almost all the rural participants had formal education and the proportion of women with formal education was significantly higher than that of men (*X*^2 ^= 4.922, p = 0.027).

**Table 1:** Socio-demographic characteristics of the participants

	population	
	
Characteristics	Forest-aboriginal (n = 100) n (%)	Rural (n = 123) n (%)
**Age**		
18-40	76 (76.0)	75 (61.0)
>40	24 (24.0)	48 (39.0)
**Sex**		
Male	38 (38.0)	46 (37.4)
Female	62 (62.0)	77 (62.6)
**Religion**		
Non-religious	99 (99.0)	0 (0.0)
Muslim	1 (1.0)	74 (60.1)
Buddhist	0 (0.0)	36 (29.3)
Hindu	0 (0.0)	13 (10.6)
**Education**		
None	38 (38.0)	6 (4.9)
Kindergarten	5 (5.0)	2 (1.6)
Primary school	49 (49.0)	57 (46.3)
Secondary school	8 (8.0)	51 (41.5)
Tertiary	0 (0.0)	5 (4.1)
University	0 (0.0)	2 (1.6)
**Occupation**		
Working	31 (31.0)	38 (30.9)
Non-working	35 (35.0)	36 (29.3)
Housewife	34 (34.0)	47 (38.2)
Students	0 (0.0)	2 (1.6)
**Races**		
Aborigine	100 (100.0)	0 (0.0)
Malay	0 (0.0)	74 (60.1)
Chinese	0 (0.0)	36 (29.3)
Indian	0 (0.0)	13 (10.6)

### Malaria knowledge

Data about the participants' knowledge and attitudes to malaria are shown in Table 2. About half of the aboriginal participants believed that malaria is transmitted by mosquito bites and this was significantly associated with the educational level (*X*^2 ^= 4.244, p = 0.039). Aboriginal participants who had previously been infected with malaria showed better knowledge of the symptoms of malaria than those with no history of infection (*X*^2 ^= 6.810, p = 0.009). On the other hand, the majority (86.2%) of the rural participants have knowledge of malaria as a disease and 70.7% of them believed that malaria is transmitted through the bite of mosquitoes and this was found to be influenced by their level of education; participants who had better education showed better knowledge about malaria as a disease and malaria symptoms (*X*^2 ^= 24.037, p < 0.001; ***X*^2 ^**= 4.416, p = 0.036 respectively). Moreover, Malay participants showed a higher level of knowledge about malaria transmission than Chinese and Indian (*X*^2 ^= 6.234, p = 0.013). The findings also showed that attitudes towards the severity of malaria were significantly higher among aboriginal participants who had previously been infected with malaria (*X*^2 ^= 4.421, p = 0.036).

**Table 2:** Participants' knowledge about malaria transmission and symptoms, and attitudes towards malaria severity

	population		
		
Variables	Forest-aboriginal (n = 100) n (%)	Rural (n = 123) n (%)	χ^2 ^significance
**Transmission of malaria**			
Mosquito bites	50 (50.0)	87 (70.7)	p < 0.01
Use of stagnant water	46 (46.0)	6 (4.9)	p < 0.001
From forest	17 (17.0)	0 (0.0)	p < 0.001
Human-to- human	7 (7.0)	4 (3.3)	ns
From weather/sun	3 (3.0)	0 (0)	ns
No knowledge	16 (16.0)	26 (21.1)	ns
**Symptoms of malaria**			
Fever	76 (76.0)	95 (77.2)	ns
Chill and rigor	56 (56.0)	27 (22.0)	p < 0.001
Headache	30 (30.0)	24 (19.5)	ns
Vomiting	11 (11.0)	16 (13.0)	ns
Body pain/weakness	10 (10.0)	7 (5.7)	ns
Loss of appetite	9 (9.0)	9 (7.3)	ns
Red rash	8 (8.0)	18 (14.6)	ns
Abdominal discomfort	2 (2.0)	0 (0.0)	ns
No knowledge	13 (13.0)	18 (14.6)	ns
**Is malaria a serious disease?**			
Yes	72 (72.0)	93 (75.6)	ns
No	10 (10.0)	13 (10.6)	
No knowledge	18 (18.0)	17 (13.8)	

### Malaria treatment-seeking behaviour and prevention

Data about treatment-seeking behaviour and prevention methods are shown in Table 3. The vast majority of the rural participants (95.1%) indicated that they would seek treatment from health centre and this was found to be associated significantly with the educational level and age of the participants (***X^2 ^***= 6.236, p = 0.013; *X*^2 ^= 9.856, p = 0.002 respectively). Similarly, the educational level, age and race of the rural participants were associated significantly with the practising of effective preventive measures (*X*^2 ^= 4.634, p = 0.031; *X*^2 ^= 5.483, p = 0.019; *X*^2 ^= 7.965, p = 0.019, respectively).

**Table 3:** Participants' practices in malaria treatment-seeking behaviour and prevention methods

	population		
		
Variables	Forest-aboriginal (n = 100) n (%)	Rural (n = 123) n (%)	χ^2 ^significance
**Treatment-seeking behaviour**			
Use herbal remedies as first line activity	28 (28.0)	19 (15.4)	p < 0.05
Witchcraft as a first line activity	13 (13.0)	0 (0.0)	p < 0.001
Go to clinic as a first line activity (within 24 hrs of fever onset)	65 (65.0)	117 (95.1)	p < 0.001
Take anti-malarial/antipyretic medicine	0 (0.0)	6 (4.9)	p < 0.05
**Malaria prevention methods**			
Use of mosquito bed nets	63 (63.0)	76 (61.8)	ns
Keep the house/surroundings clean	30 (30.0)	46 (37.4)	ns
Elimination of breeding sites	0 (0.0)	8 (6.5)	p < 0.001
Use of insecticide/spraying	3 (3.0)	33 (26.8)	p < 0.001
Fumigation by smoke	7 (7.0)	28 (22.8)	p < 0.01
Use of anti-malarials	1 (1.0)	8 (6.5)	p < 0.05
Use of medicinal plants	10 (10.0)	6 (4.9)	ns
No knowledge	21 (21.0)	23 (18.7)	ns

Overall, knowledge about malaria and its transmission was significantly higher among the rural participants than the Aborigines (*X*^2 ^= 6.746, p = 0.009; *X*^2 ^= 10.006, p = 0.002, respectively) (Table 2). Similarly, the use of medicinal plants and beliefs in witchcraft and sorcery in treating febrile diseases were significantly higher among the aboriginal population (*X*^2 ^= 5.225, p = 0.022; *X*^2 ^= 16.980, p < 0.001, respectively) (Table 3). On the other hand, there was no significant difference between the two communities in terms of the knowledge about malaria symptoms, their attitude towards its severity and the use of mosquito bed nets. However, the knowledge and practice of different preventive measures to combat malaria such as insecticides and the elimination of breeding areas was significantly higher among the rural population than the aborigines (*X*^2 ^= 23.136, p < 0.001).

## Discussion

This study is the first to investigate the KAP on malaria in Peninsular Malaysia, which is directly essential to enhance community awareness of malaria. Findings of the present study indicated that there were significant differences between the aboriginal and rural communities in terms of their knowledge of malaria transmission, practices in treatment-seeking behaviour and their understanding of effective preventive measures. This could be explained by the better educational level and the higher number of health facilities reported among the rural population. In addition, cultural beliefs and traditions about diseases among the aboriginal population in the remote areas may contribute considerably to their poor knowledge of malaria. Generally, aboriginal people attribute diseases to ghosts and evil spirits, and this study found that more than one third of the sick aboriginal people normally consulted herbalists or sorcerers who provided remedies and rituals to fight the evil spirits.

The present study also reported many mistaken beliefs about the disease particularly in the aboriginal areas where a large number of the participants demonstrated a misconception about the transmission of malaria. This emphasizes the need for effective intervention to improve the level of knowledge in this community. A noteworthy number of participants believed that malaria is transmitted by stagnant water, walking in the forest, from human to human and nor others. Although most of the participants associated malaria with mosquito bites, none of them knew that *Plasmodium *is the causative agent responsible for malaria nor how mosquitoes acquire the parasite. The role of mosquitoes in malaria transmission was known to 50% and 70.7% of the aboriginal and rural participants, respectively. This figure was lower than that reported in Swaziland (Southern Africa), a country earmarked for malaria elimination [6]. Accordingly, it is clear that the lack of knowledge, among the target population, about the aetiology of the disease as well as the role of mosquitoes in causing malaria may create an additional burden and costs for controlling the disease and may cause the failure of the malaria elimination programme.

Education plays an important role in people's perceptions and practices of treating and controlling malaria. Previous studies from Africa showed a positive correlation between the number of years of formal education and the perception of environmental management and use of bed nets to prevent malaria, and use of chloroquine to treat malaria [7,8]. Moreover, as the number of years of formal education increased the perception that herbal medicine could be used to treat malaria decreased [8]. In harmony with these findings, the present study showed that the better educational level of the rural community reflected a better knowledge of practices in malaria treatment and prevention than the aboriginal community. In addition, the level of education showed a significant impact on the population's KAP on malaria at the community level.

The findings of this study showed that most people in both communities had information about the symptoms of malaria and more than three-quarters of the participants recognized fever, chills/rigors and headache as most common symptoms. This was in agreement with the previous studies in tropical and subtropical malaria endemic countries [9,10]. It should be noted that a considerable number of participants in both communities showed confusion between the symptoms of malaria and dengue fever which is also endemic in these areas. However, this may not affect the disease control in terms of mosquito control, but it may lead to serious complications in patients who believe that dengue fever can be cured by taking antipyretics and drinking fluids [11].

Promising results about treatment-seeking behaviour were reported; almost all the rural participants and two-thirds of the aboriginal participants seek treatment at health centres within 24 hours of the onset of symptoms. Previous studies in rural areas in Southeast Asia showed that more than half of the population opts for self-treatment without visiting a health facility [12,13]. The better behaviour reported by the present study could be due to the availability of health facilities and access to their services to all Malaysians throughout the country. Most of the aboriginal people who use medicinal plants and believe in witchcraft as a treatment for febrile diseases go to health centres for treatment, either within 24 hours while at the same resorting to those exercising those practices to support the modern treatment or within 48-72 hours waiting for the outcome of traditional recipes for a short period of time and then seeking treatment from the health facilities. Waiting until the disease has worsened, and a belief that febrile diseases resolve spontaneously are among the reasons for not seeking modern treatment on the day of fever onset [14].

Regarding the understanding of the measures for the prevention of malaria, the present study showed that most of the participants were aware that malaria can be prevented. However, many misconceptions about malaria prevention measures were reported. In both communities, more than one-third of the participants do not use bed nets to prevent mosquito bites. This could be due to the reliance of these populations on the government in fighting the disease without taking enough personal precautions. The poor usage of mosquito bed nets might also be attributed to the cost and to the lack of knowledge that mosquitoes are the causative agents of malaria [15,16]. The comparison between the forest-aboriginal and rural communities is of great benefit to determine the nature of the adaptations required in the plan for future intervention. Public awareness programmes to promote a better understanding of malaria transmission and the active participation of the aboriginal communities in malaria control activities are deemed necessary. Such awareness and participation will bring about positive changes and adaptation in their cultural beliefs and practices and this can help in reducing the incidence of malaria.

## Conclusions

This study revealed that knowledge about the transmission and prevention of malaria is inadequate, in particular, among the aboriginal population and this could be a challenging obstacle to the elimination of malaria from Malaysia. Hence, a malaria elimination programme should take into consideration that health education and community mobilization will enhance prevention and instil better knowledge of malaria transmission and prevention. Accordingly, the findings of this survey will assist the health authorities to establish clearer knowledge, attitudes and practices of the population, with regard to malaria, enabling the use of efficient tools for health education and improving and sustaining good practices for malaria prevention.

## Competing interests

The authors declare that they have no competing interests.

## Authors' contributions

AHA was involved in all phases of the study, including study design, data collection data analysis, interpretation, and the write-up of the manuscript; ZMN and RM designed and supervised the study, and revised the manuscript. HMA was involved in the collection and statistical analysis of data and the critical revision of the manuscript for publication. All authors read and approved the final manuscript.
